# Analysis of polymorphisms in *Plasmodium falciparum* genes related to drug resistance: a survey over four decades under different treatment policies in Brazil

**DOI:** 10.1186/1475-2875-13-372

**Published:** 2014-09-19

**Authors:** Juliana Inoue, Dinora Lopes, Virgílio do Rosário, Marta Machado, Angélica D Hristov, Giselle FMC Lima, Maria J Costa-Nascimento, Aluísio C Segurado, Silvia M Di Santi

**Affiliations:** Departamento de Moléstias Infecciosas e Parasitárias, Faculdade de Medicina, Universidade de São Paulo, Av. Dr. Enéas Carvalho de Aguiar, 470 sala 107, 05403-000 São Paulo, SP Brazil; Instituto de Higiene e Medicina Tropical, Universidade Nova de Lisboa, Rua da Junqueira, 100, 1349-008 Lisboa, Portugal; Núcleo de Estudos em Malária, Superintendência de Controle de Endemias/Instituto de Medicina Tropical de São Paulo, Universidade de São Paulo, Av. Dr. Enéas Carvalho de Aguiar, 470 sala 107, 05403-000 São Paulo, SP Brazil

**Keywords:** Anti-malarial resistance, Molecular markers, *Plasmodium falciparum*, *pfcrt*, *pfmdr1*, *pfdhfr*, *pfdhps*

## Abstract

**Background:**

Anti-malarial resistance in *Plasmodium falciparum* remains an obstacle for malaria control. Resistance-associated genes were analysed in Brazilian samples over four decades to evaluate the impact of different treatment regimens on the parasite genetic profile.

**Methods:**

Samples were collected on filter paper from patients infected in the Amazon region from 1984 to 2011. DNA was extracted with Chelex® 100 and monoinfection confirmed by PCR. SNPs in the *pfcrt*, *pfmdr1, pfdhfr* and *pfdhps* genes were assessed by PCR-RFLP. The *pfmdr1* copy number was estimated using real time quantitative PCR with SYBR® Green. Parasite response was assessed *ex vivo* with seven concentrations of each anti-malarial. Patients were treated according to Brazilian guidelines: quinine plus tetracycline or mefloquine in period 1 and ACT in period 2.

**Results:**

All 96 samples presented the *pfcrt* 76T mutant throughout the assessed periods. In addition, all isolates showed *ex vivo* chloroquine resistance. The *pfmdr1* 86Y was detected in 1.5% of samples in period 1, and in 25% in period 2. All samples presented the *pfmdr1* 1246Y. The analysis of *pfmdr1* copy number showed amplification in 37.3% in period 1 and in 42% in period 2. Mutations in *pfdhfr* were shown as follows: 51I in all samples in period 1 and in 81.2% in period 2; 59R in 6.4% in period 2. The *pfdhfr* 108N and the *pfdhps* 437G were seen in all samples along time; the *pfdhps* 540E in 93.7% in period 1 and in 75% in period 2.

**Conclusions:**

The 76T mutation associated to chloroquine resistance is still present in the parasite population, although this anti-malarial was withdrawn from the chemotherapy of *P. falciparum* in Brazil in the mid-1980s. All isolates assayed *ex vivo* for chloroquine showed resistant phenotype and 76T. No association was observed between *pfmdr1* mutations and resistance to quinine, mefloquine and artemisinin derivatives. Additionally, the *pfdhfr* 108N mutation was detected in all samples throughout the evaluated periods, demonstrating fixation of the mutant allele in the parasite population. Changes in Brazilian national guidelines for the malaria chemotherapy in the last 27 years yielded a discreet genetic impact in the parasite population.

## Background

Malaria remains a major public health concern, with the occurrence of about 207 million cases and accounting for 627,000 deaths worldwide in 2012 [1]. In Brazil 177,767 malaria cases were reported in 2013, 99.6% of which were acquired in the Amazon region, the main endemic area for the disease in South America. In terms of species distribution, *Plasmodium vivax* accounted for 82% of Brazilian malaria cases, followed by *Plasmodium falciparum* (16.5%) and mixed infections (1.5%) [2]. Previously, there has been higher prevalence of *P. falciparum* [1]. However, *P. falciparum* is known to cause most complicated clinical cases and to have become resistant to almost all anti-malarial drugs.

Chloroquine-resistant *P. falciparum* was initially reported in Brazil in the 1960s [3] and thereafter in other South American countries [4]. Further similar cases were reported in Brazil in the 1980s in the states of Amazonas and Maranhão [5, 6]. For this reason, an alternative therapeutic regimen that combined sulphadoxine and pyrimethamine (SP) was adopted as the first line treatment recommendation. However, resistance to SP was reported in northern Tocantins State soon after its use was implemented [7]. In 1982, resistance to SP was also described in other Brazilian states, such as Rondônia, Pará, Amazonas and Mato Grosso [8, 9]. Subsequently, Boulos *et al.* [10] reported 100% of therapeutic failure to SP in 54 patients, and in the 1980s quinine in combination with tetracycline was introduced as first-line treatment. Mefloquine was incorporated in the 1990s as a new tool to treat resistant *P. falciparum* malaria*,* but the emergence of chemoresistance [11] led to the adoption of new strategies, such as artemisinin-based combination therapy (ACT), as recommended by the WHO in order to refrain the spread of resistance [12]. In Brazil, since 2006 the first-line treatment recommendation for non-complicated *P. falciparum* infections is the use of artemether plus lumefantrine or artesunate plus mefloquine combinations [13]. Nevertheless, decreased sensitivity has already been reported in Southeast Asia [14–16], Africa [17] and South America [18].

Resistance to anti-malarials is caused mainly by drug pressure and selection of mutated parasites, and in most cases single nucleotide polymorphisms (SNPs) in the parasite genome are involved. Resistance to chloroquine has been shown associated to SNPs in the *P. falciparum* chloroquine resistant transporter gene (*pfcrt*) on chromosome 7. Substitution of a lysine by threonine at codon 76 was found in resistant parasites and, therefore, this mutation has been used as a molecular marker for chloroquine resistance [19, 20] for both *in vitro* and clinical resistance assessment [21]. Substitution of amino acids (T, N or I) in the *pfcrt* mutant alters the electric charge of the membrane, leading to chloroquine efflux from the digestive vacuole [22, 19].

The *P. falciparum* multidrug resistant gene (*pfmdr1*) on chromosome 5 encodes the P-glycoprotein homologue-1 located in the parasite food vacuole and it is believed to be associated to resistance to several anti-malarials [23]. Substitution of asparagine by tyrosine at codon 86 has been shown associated with chloroquine and quinine resistance, and increased sensitivity to mefloquine and artemisinin derivatives [24]. Reed *et al.* [23] suggested that amino acid change in position 1246 influences response to mefloquine, quinine, halofantrine and artemisinin and modulates sensitivity to chloroquine. Price *et al.* [25] reported an association between increased *pfmdr1* copy number and risk of clinical failure with mefloquine when used in monotherapy or in combination with artesunate. Parasites with increased *pfmdr1* copy number presented higher median IC_50_ values of mefloquine, artemisinin derivatives, quinine and lumefantrine [25–27]. However, some authors did not find a correlation between increased *pfmdr1* copy numbers and mefloquine resistance [28, 29].

Pyrimethamine and sulphadoxine are antifolate drugs that act by inhibiting the dihydrofolate reductase and dihydropteroate synthase enzymes, respectively. Pyrimethamine resistant *P. falciparum* presents a substitution of serine by asparagine at codon 108 of the *pfdhfr* gene that encodes the dihydrofolate reductase enzyme. Additionally, resistant parasites may present N51I and C59R mutations in this gene [30, 31]. The A437G mutation in the *P. falciparum* dihydropteroate synthase (*pfdhps)* gene confers resistance to sulphadoxine. Additionally, other mutations at codons 436, 540, 581, and 613 are responsible for high level of resistance [32]. The quintuple mutation (108N/51I/59R and 437G/540E) in these two genes is considered a marker of resistance to SP [33].

The continuous monitoring of molecular markers is a very useful tool for malaria control, since it may guide therapeutic policies. Several studies have assessed allele frequencies in different time periods in Africa and Asia [34, 35]. In some scenarios, changes in chemotherapy guidelines have led to a decline in the frequency of mutant alleles after drug pressure was withdrawn [36]. In this sense, it is important to investigate the genotypic resistance profile of parasite populations submitted to different drug pressures along time when different therapeutic regimens were employed. This survey is of particular importance for Brazilian isolates monitoring since this country accounts for 52% of reported malaria cases in the Americas [1]. This study aimed at describing the occurrence of mutations in the *pfcrt*, *pfmdr1, pfdhfr* and *pfdhps* genes over four decades, when the parasite population was exposed to selective pressure of various therapeutic approaches.

## Methods

### Study setting and ethics

**Figure 1:**
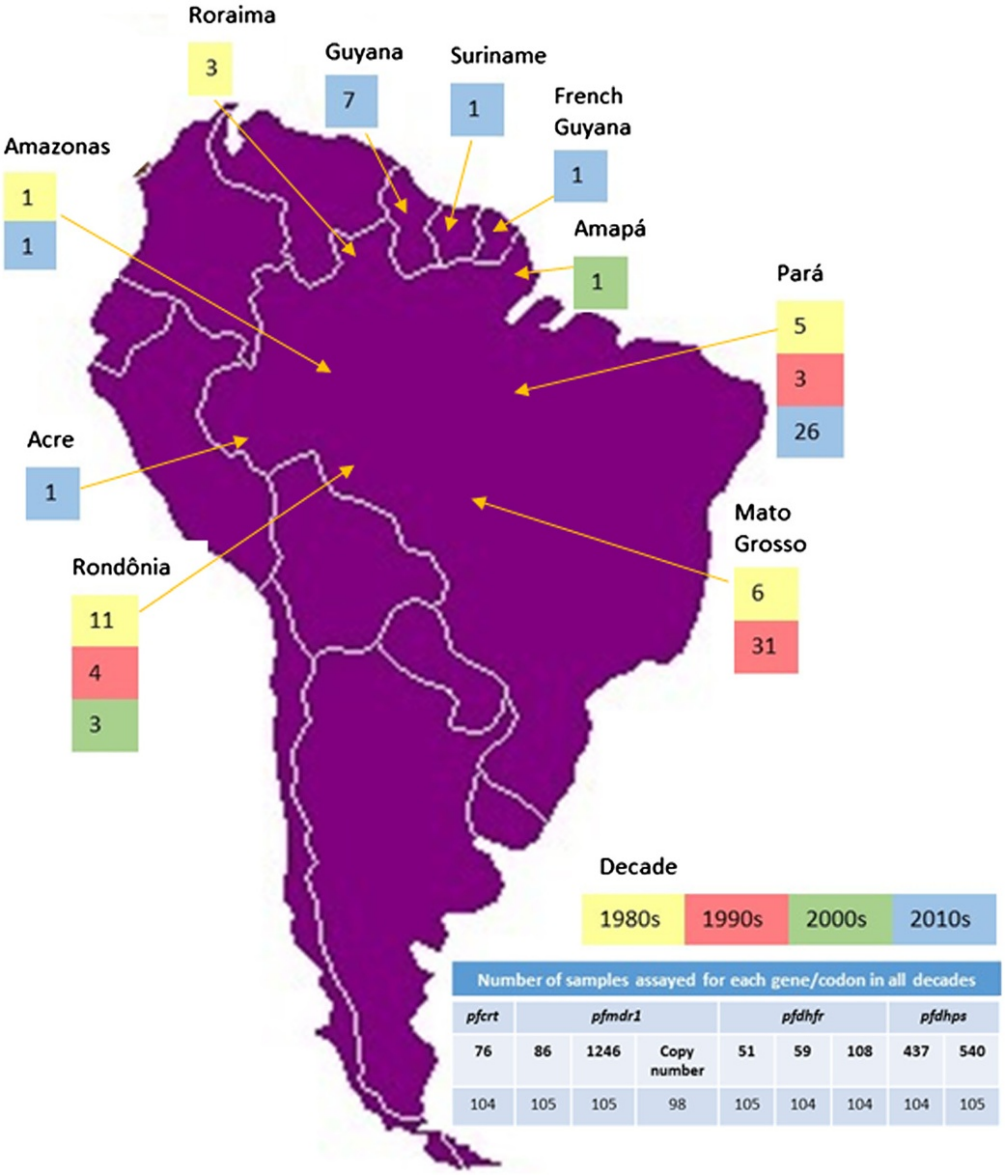
**Distribution of** ***P. falciparum*** **samples collected from 1984 to 2011 according to site of infection.** In the detail, the number of samples assayed for *pfcrt*, *pfmdr1*, *pfdhfr* and *pfdhps*.

Samples were collected from patients admitted to health services in São Paulo, SP and Santarém and Alenquer, PA, with clinical malaria acquired in the Brazilian states of Acre, Amapá, Amazonas, Mato Grosso, Pará, Rondônia and Roraima. These states are located in the Amazon basin, northern Brazil, where malaria transmission occurs mainly during the dry season. Additionally, samples from subjects infected in French Guiana, Guyana and Suriname, countries bordering Brazil, were included (Figure 1). Most of the latter cases were seen in Brazilian miners who worked in bordering countries and returned to Brazil for diagnosis and treatment. The number of samples from each decade reflects epidemiological aspects of malaria in Brazil, with fluctuations in the incidence of the disease over the years, as influenced by the implementation of agricultural settlement projects and gold mining activities [37]. Ethical approval for the study was obtained from CONEP/Ministry of Health, Brazil (206/2012), and all subjects gave informed consent for participation and sample collection.

### Study population and blood sample collection

Inclusion criteria required *P. falciparum* single infection diagnosed by microscopy with any parasitaemia in subjects of any age. Samples were collected from 1984 to 2011. Samples obtained from 1984 to 2009 were stored in the Malaria Laboratory/SUCEN/IMTSP biobank in liquid Nitrogen. After thawing, 50 μL were plotted on Whatman 3® filter paper (Sigma-Aldrich®, St. Louis, MO, USA) in duplicate. Samples from 2010 and 2011 were collected directly on filter paper by finger printing and stored at room temperature until use. Although samples are presented by decade of collection, the study population was analysed according to the Brazilian national therapeutic guidelines for malaria chemotherapy in use in the first two decades (quinine plus tetracycline or mefloquine) and in the last two decades (ACT), named periods 1 and 2, respectively.

### Molecular identification of *Plasmodium*species

DNA from filter paper was extracted with Chelex® 100 (Bio-Rad™, Hercules, CA, USA) according to the protocol by Plowe *et al.* [38] and stored at -20°C until use. To confirm single infection by *P. falciparum,* nested PCR targeting ssrRNA genes was performed following published protocols [39].

### Detection of *pfcrt, pfmdr1, pfdhfr*and *pfdhps*genotypic polymorphisms

PCR followed by restriction fragment length polymorphism analysis (RFLP) was performed to search for SNPs at codons 76 of *pfcrt*; 86 and 1246 of *pfmdr1*; 51, 59 and 108 of *pfdhfr,* and 437 and 540 of *pfdhps* genes. For *pfcrt* 76 and *pfmdr1* 86, fragments of 206 and 504 bp, respectively, were amplified by PCR and submitted to enzyme digestion with *ApoI*. For *pfmdr1* 1246, a 508 bp fragment was amplified and digested with *Eco321*. Fragments of 206, 326 and 522 bp were amplified for codons 51, 59 and 108 of *pfdhfr* and of 438 bp for both 437 and 540 codons of *pfdhps* gene. Digestion was performed with *TasI*, *PdmI*, *AluI* for codons 51, 59 and 108 of *pfdhfr* and with *HpyF10VI* and *BsegI* for codons 437 and 540 of *pfdhps* gene. DNA samples from *P. falciparum* clones 3D7, Dd2 and Hb3 were used as controls. After RFLP, digestion products were loaded onto ethidium bromide-stained 1× TBE 2% agarose gels, and fragment sizes measured visually based on a 100 bp DNA ladder and compared to control fragments [40–42].

### Determination of *pfmdr1*copy number

Real time quantitative PCR was performed with iQ™ SYBR® Green Supermix (Bio Rad™, Hercules, CA, USA) to determine *pfmdr1* gene copy number. Reactions were assayed in iCycler iQ Multicolor Real-Time PCR Detection System (Bio Rad™, Hercules, CA, USA), with a 10-minute incubation at 95°C, followed by 40 cycles with 15 seconds 95°C/1 minute 60°C. The melting curve was obtained with one 10-minute cycle at 95°C. Samples were assayed in triplicate, following a published protocol [43]. The 2^-ΔΔCt^ method was applied to estimate the *pfmdr1* copy number, using DNA of two calibrators with known copy numbers (3D7, harbouring one copy and Dd2, four copies). The housekeeping gene *pfβactin1* was used as recommended in order to normalize data.

### *Ex vivo*anti-malarial sensitivity test

Twenty-two samples were assayed by *ex vivo* chemosensitivity tests. Samples were collected in São Paulo, regardless of site of malaria acquisition, during the two first decades, period when quinine plus tetracycline and mefloquine were employed. Samples from patients who received any anti-malarial drug in the previous 30 days before sample collection were excluded. Each sample was assayed with chloroquine, quinine and mefloquine to determine Minimal Inhibitory Concentrations (MIC). Isolates were exposed to different drug concentrations in serial dilutions for 72 hours in pre-dosed microplates, as follows: 10, 20, 40, 57, 80, 160 and 320 nM chloroquine, 40, 80, 160, 320, 640, 1280 and 2560 nM quinine and 5, 10, 20, 40, 57, 80 and 160 nM mefloquine. Infected blood was added to RPMI complete medium in 10% hematocrit, in 5% CO_2_, 5% O_2_ and 90% N_2_ atmosphere in a 37°C incubator. Drug response was assessed counting the number of schizonts/200 parasites in Giemsa-stained thick blood films, using microcultures carried out in the same conditions but without exposure to anti-malarial drugs as controls. In addition, reference *P. falciparum* Palo Alto and K1 isolates were used as controls for chloroquine-sensitive and -resistant strains, respectively [44].

### *In vivo*treatment

**Figure 2:**
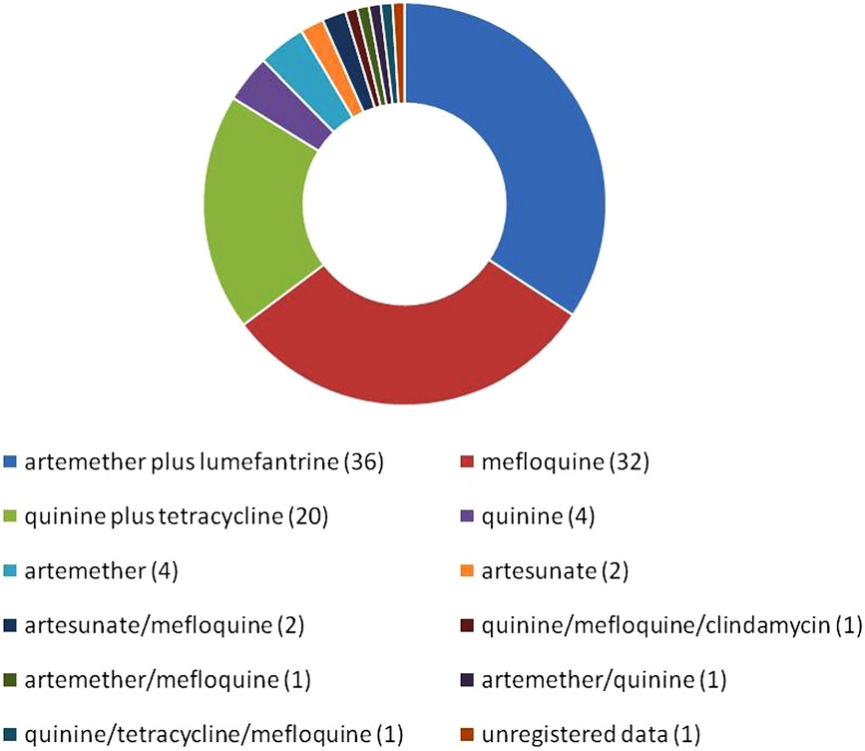
**Therapeutic schemes in patients treated from 1984 to 2011 based on Brazilian guidelines or approved therapeutic protocols.**

Clinical data related to treatment were obtained from medical records both at the time of diagnosis and during follow-up. All patients were treated following Brazilian national guidelines for malaria chemotherapy or approved research therapeutic protocols (artemether or artesunate in monotherapy). Recrudescent parasitaemia after use of artemisinin derivatives in monotherapy were treated according to Brazilian guidelines [13, 45]. Monitoring of parasitaemia was carried out on days 3, 7, 14, 28 and 35 after treatment was started. Therapeutic regimens are presented in Figure 2.

### Statistical analysis

The Fisher’s Exact Test (GraphPad) was applied in search of associations between *in vivo* or *ex vivo* phenotypic responses to quinine, mefloquine and artemisinin derivatives and the presence of mutations at each codon (*pfmdr1* 86 and 1246). The same test was used to check for association between phenotypic response and *pfmdr1* copy number. P values <0.05 were considered statistically significant.

## Results

### Origin of samples

A total of 96 samples from patients infected in Brazil were assessed. Twenty-six samples were collected in the 1980s, 38 in the 1990s, four in the 2000s and 28 in the 2010s. Additionally, nine samples collected from patients infected in the 2010s in countries bordering Brazil (French Guiana, Guyana and Suriname) were evaluated.

### *pfcrt*and *pfmdr1*

All Brazilian samples presented the *pfcrt* 76T mutant in periods 1 and 2. As for *pfmdr1*, the 86Y mutant was identified through in the 1990s and 2010s in different frequencies, when all but one mutant isolate harbouring the genotype 86Y were obtained from patients diagnosed in Pará State (Table 1). This mutant was also observed in 43% of samples collected in Pará State from patients infected in Guyana in the 2010s. All Brazilian samples as well as isolates from bordering countries presented the 1246Y mutant genotype in the two evaluated periods (Table 1). Assessment of *pfmdr1* copy numbers from Brazilian isolates are summarized in Table 1.

**Table 1:** **Genotyping of** ***pfcrt*** **,** ***pfmdr1*** **,** ***pfdhfr*** **and** ***pfdhps*** **in** ***P. falciparum*** **from Brazil, according to two distinct therapeutic regimen periods**

Period	***pfcrt***	***pfmdr1***				***pfdhfr***					***pfdhps***		
	76	86	1246	Copy number		51		59		108	437	540	
	T (%)	Y (%)	Y (%)	1 (%)	>1 (%)	I (%)	N + I (%)	R (%)	C + R (%)	N (%)	G (%)	E (%)	K + E (%)
1	100	1.5	100	62.7	37.3	100	0	0	0	100	100	93.7	1.5
2	100	25	100	58	42	81.2	18.7	6.4	16.1	100	100	75	0

### *pfdhfr*and *pfdhps*

Mutations in positions 51, 59 and 108 of the *pfdhfr* gene are described in Table 1. Mutants at position 51 are less frequently described in period 2 and at position 59 emerged as single or mixed infections. All isolates presented the mutation in position 108 in all periods. Considering the samples from countries bordering Brazil, 8/9 presented 51I, 1/9 51 N + I and 1/9 59 C + R; all samples presented 108 N genotype. With respect to *pfdhps* gene, samples from both periods presented the 437G mutant genotype. At position 540, a slight decrease in the frequency of the mutant was observed (Table 1). Regarding the samples from countries bordering Brazil, all samples presented 437G and 540E.

### Multiple mutants

**Figure 3:**
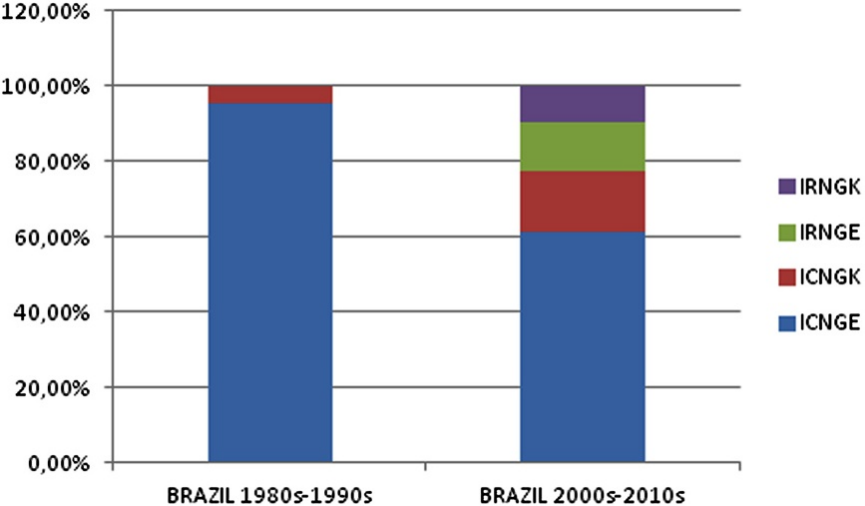
**Distribution of quintuple (IRNGE), quadruple (ICNGE and IRNGK) and triple (ICNGK) mutations in** ***pfdhfr*** **and** ***pfdhps*** **genes in Brazilian isolates.**

As for multiple mutations in positions 51, 59 and 108 of the *pfdhfr* gene and 437 and 540 of *pfdhps*, 95.3% and 4.7% of Brazilian samples from period 1 presented quadruple (ICNGE) and triple mutation (ICNGK), respectively. In period 2, it was detected quintuple mutation (IRNGE) in 12.9%, quadruple ICNGE in 61.3% and IRNGK in 9.7%. Triple mutation (ICNGK) was detected in 16.1%, as shown in Figure 3.

### Clinical response to chemotherapy

**Figure 4:**
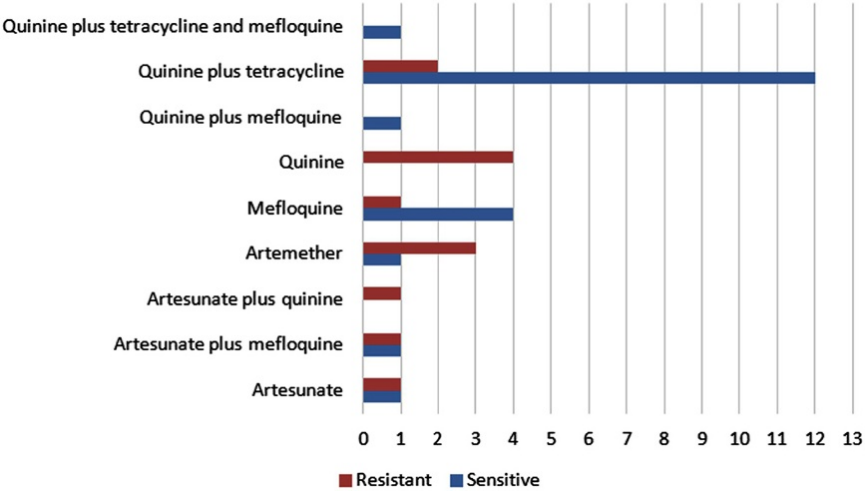
**Treatment response according to therapeutic regimens in patients with at least 28-day follow-up.**

Thirty-four patients who completed clinical follow-up for at least 28 days after being started on chemotherapy were evaluated. Out of these 27 had received anti-malarials following Brazilian national guidelines at time of diagnosis, six patients had been treated with artemisinin derivatives in monotherapy as subjects enrolled for a clinical research protocol, and one individual was treated with artesunate plus quinine hydrochloride due to very high parasitaemia at time of diagnosis. Patients were defined as harbouring sensitive or resistant parasites in case they exhibited absence or presence, respectively, of *Plasmodium* on day 28 after being started on chemotherapy. Clinical response to treatment according to therapeutic regimens is shown in Figure 4.

### *pfcrt*genotypic polymorphism versus *ex vivo*response to chloroquine

Twenty-one samples were assayed for chloroquine sensitivity using an *ex vivo* test. All isolates showed a resistant phenotype with MIC ≥ 80 nM. In addition, all samples showed the *pfcrt* 76T mutant genotype when assayed by RFLP.

### *pfmdr1*genotypic polymorphism versus *ex vivo*and *in vivo*responses

Among the isolates resistant to chloroquine, one was resistant to quinine but not to mefloquine, and six were resistant to mefloquine but not to quinine. Nineteen samples were tested *ex vivo* for quinine sensitivity, 5.2% of which revealed a resistant phenotype, with MIC > 2560 nM. On molecular analysis, this sample presented a wild type genotype for codon 86 and a 1246Y mutant. All sensitive samples presented the same genotypic profile. *Ex vivo* tests for mefloquine were performed in 22 samples and in 27.3% of them yielded MIC > 160 nM. The wild type genotype for codon 86 and the mutant 1246Y were seen in all resistant samples. One sensitive sample showed the mutant 86Y. Regarding copy numbers, out of six mefloquine-resistant isolates, four presented two copies of *pfmdr1* and two, one copy only. Five out of 13 mefloquine-sensitive isolates showed two or more gene copies and eight out of 13, one copy only. Three isolates assayed for mefloquine showed no amplification for *pfmdr1* copy number assessment.

As far as the *in vivo* response to chemotherapy is concerned, all patients presented a clinical recrudescence after having received quinine in monotherapy, 20% after treatment with mefloquine and 66.6% of those who were given artemisinin derivatives in monotherapy. All these samples harboured the N86 wild genotype and the 1246Y mutant as well as those from patients who did not present recrudescent malaria.

### Statistical analysis

The Fisher’s Exact Test showed no association between the *in vivo* clinical response to quinine and the occurrence of *pfmdr1* mutations in codons 86 and 1246 (p = 1.000), neither between clinical response after chemotherapy and the *pfmdr1* copy number (p = 1.000). In addition, no association was found between the *in vivo* response to mefloquine and mutations in codon 86 (p = 1.000), 1246 (p = 1.000) or with *pfmdr1* copy number (p = 1.000). Likewise, mutations in codons 86 and 1246 or variation in gene copy number were not shown associated with *in vivo* clinical response to artemisinin derivatives (p = 1.000). No statistically significant association was seen between the *ex vivo* response to quinine and mefloquine and *pfmdr1* mutations in codons 86 and 1246 (p = 1.000), neither between copy number and *ex vivo* clinical response to mefloquine (p = 0.3498) or quinine (p = 1.000).

## Discussion

This is the first study to assess *P. falciparum* genotypic polymorphisms related to chemoresistance in Brazilian samples collected over a 27-year period when different national therapeutic policies were successively in place. Analysis of *pfcrt* gene showed that all samples harboured the 76T mutation, strongly associated to chloroquine resistance, throughout the evaluated periods, even though this 4-aminoquinoline was replaced by quinine in monotherapy or associated to tetracycline in Brazilian national guidelines for malaria chemotherapy in the mid-1980s [46, 47]. Assessment of *pfcrt* mutations is relevant to monitor molecular changes that could have led to reversal of chloroquine resistance after drug withdrawal, as seen in Malawi. In this African country, absence of drug pressure yielded restauration of chloroquine-sensitive genotypes in the parasite population [36]. A similar phenomenon occurred in Kenya with substantial reversal to the K76 genotype [48]. In Africa, *P. falciparum* accounts for most human malaria infections and chloroquine withdrawal after introduction of ACT removed selective drug pressure on the parasite. Such reversal, however, is not expected to take place in South America, a region characterized by high prevalence of *P. vivax* infections, which require uninterrupted use of chloroquine. In such a scenario, fixation of the mutant genotype is supported. In addition, it is important to consider that chloroquine is still widely used in this region for the treatment of rheumatic diseases including systemic lupus erythematosus, contributing to an increased drug pressure in the *P. falciparum* genome [49]. The results of *pfcrt* genotyping with samples from countries bordering Brazil are in agreement with studies conducted in French Guiana [50] and Suriname [51], where therapeutic policies have been similar to those adopted in Brazil. Moreover, the molecular results are concordant with results of the *ex vivo* assays that revealed full resistance to chloroquine with all tested isolates.

The *pfmdr1* gene has been previously reported to be involved with increased resistance to chloroquine and quinine, and with increased sensitivity to mefloquine and artemisinins [24] or, in contrast, with increased sensitivity to quinine [52] *.* In this study, the N86 wild type was detected in almost all samples from periods 1 and 2, in agreement with earlier reports from Suriname [51] and other South American countries [53, 54]. Considering that in period 1 Brazilian national chemotherapy guidelines recommended three different therapeutic approaches, i.e., use of chloroquine, quinine and mefloquine, it is possible to conclude that drug pressure was not able to select a mutant genotype. This hypothesis is corroborated by the lack of association between results obtained in *in vivo* and *ex vivo* assays. However, an aspect to be highlighted is the emergence of the 86Y mutant in the last decade of period 2 in 25% of Brazilian samples and in 43% of isolates from Guyana. Even though increased sensitivity to artemether-lumefantrine has been linked to the *pfmdr1* N86 wild type in Africa [55], Bustamante *et al*. [56] found association between decreased sensitivity to artemether and the occurrence of the 86Y mutant using *in vitro* tests. ACT was introduced in Brazil and in Guyana in 2006. In Guyana, *P. falciparum* is the most prevalent species and a drug efficacy study showed 70.1% of patients positive at day 3 after artemether-lumefantrine treatment [57]. In gold mining and logging areas in Guyana, symptoms related to malaria are first taken care of at informal health facilities, where a wide range of anti-malarials, including artemisinin derivatives is available [58, 59]. Taking the high flow of miners between Brazil and neighbouring countries into account, it is possible to hypothesize that the emergence of *pfmdr1* 86Y could be related to the indiscriminate use of ACT from the mid-2000s in that region. Although no association was found between *in vivo* failure to artemisinin and the 86Y polymorphism, selection for this mutant requires continuous monitoring of *P. falciparum* in this region. Another relevant issue to be discussed is the fact that all samples harboured the 1246Y genotype throughout the evaluated periods. These results are concordant with observations by other investigators from South America, in which analysis of this codon showed only mutant parasites [51, 54]. In conclusion, despite several changes in recommendations for malaria chemotherapy along the two periods, no genotypic selection occurred at this codon, as postulated in a study with samples from Venezuela [54].

Analysis of the *pfdhfr* gene showed the emergence of the 59R mutant in the more recent period. Additionally a high frequency of mutants at codons 51 and 108 was detected in all assessed periods. As for the *pfdhps* gene, a high frequency of mutants at codons 437 and 540 was observed. In Peru, removal of SP from chemotherapy recommendations was followed by reduction from 47% to 16.9% in the prevalence of the *pfdhfr* mutant haplotype, as well as in *pfdhps* with 83.8% of isolates exhibiting the wild type allele [60]. In contrast, a study conducted in Venezuela eight years after the withdrawal of SP showed resistance-associated mutations fixed in the parasite population, suggesting that in low transmission areas, fixation of resistant parasites is stable even after removal of drug pressure [61]. This scenario seems to occur in Brazil, where withdrawal of SP occurred in the 1980s. Although the emergence of codon 51 wild type allele was observed, the 108N mutant is still present in the parasite population. When compared to a previous study carried out in 1991 with *P. falciparum* samples from the Amazon region, results of the present study show an increase of 90% to 100% in the prevalence of the *pfdhfr* 108N mutation [62]. Such evidence discourages recommending the use of artemisinin derivatives combined with SP in Brazil. In fact, the use of artesunate plus SP in Mozambique led to a dramatic increase of mutants in a short period after the establishment of this policy [63].

Finally, all samples presented the *pfcrt* 76T genotype and resistance to chloroquine in those assayed *ex vivo*. A comparison of these results with an *in vivo* response was however unfeasible, as chloroquine has not been used in Brazil to treat falciparum malaria since the beginning of the first period. Nevertheless, the use of *pfcrt* as a molecular marker for chloroquine resistance is strengthened by the present findings. Although amplification of *pfmdr1* has been previously associated with reduced susceptibility to mefloquine [25] and lumefantrine [64] and susceptibility to quinine [27], statistical analysis in this study did not show association between mutations in *pfmdr1* codons 86 and 1246, or between *pfmdr1* copy number variation with *in vivo* or *ex vivo* responses to quinine and mefloquine. The same result was observed with the *in vivo* response to artemisinin derivatives, in a group of patients treated outside the transmission area, without any possibility of reinfection, and followed for at least 28 days. Likewise no significant differences between *ex vivo* response to dihydroartemisinin, quinine and mefloquine were observed regarding *pfmdr1* 86 and 1246 mutations, in an African study [65]. The role of *pfmdr1* in anti-malarial resistance remains thus controversial, since various epidemiological variables may differently modulate selection to which parasite populations are submitted to [66]. Furthermore, the Kelch13 gene, located on *P. falciparum* chromosome 13 [67] and the *pfAP2μ* [68] is believed to be involved in decreased response to artemisinin derivatives.

Limitations of this study include small sample sizes for *in vivo* and *ex vivo* analyses, as well the fact that the number of samples was different in periods 1 and 2. Nevertheless, the present report has been able to provide an idea of the extent in which different malaria chemotherapeutic recommendations used in Brazil have impacted *P. falciparum* genotypic profile in the country.

## Conclusions

The *pfcrt* 76T mutant was demonstrated in all analysed isolates and full chemoresistance to chloroquine was shown in *ex vivo* response assessment. The results are also conclusive with respect to the high prevalence of *pfdhfr* and *pfdhps* mutants, indicating that SP should not be combined with artemisinin derivatives in Brazil. These findings reinforce the multidrug resistant pattern of Brazilian *P. falciparum* isolates. It was confirmed the fixation of 76T *pfcrt*, in spite of the fact that chloroquine was withdrawn from chemotherapy in the beginning of the first period considered in this study. Fixation of the mutant is believed to be due to continuous use of chloroquine for *P. vivax* infections, the most prevalent human malaria in Brazil. Likewise, mutations in *pfdhfr* and *pfdhps* genes showed no remarkable changes even after substitution of SP by different treatment regimens. *Pfmdr1* presented single or multiple copies regardless of treatment policy. Analysis of SNPs in *pfmdr1* showed maintenance of the molecular profile except for emergence of 86Y in period 2, when ACT was adopted. The lack of association between phenotypic response to quinine, mefloquine and artemisinin derivatives with SNPs in the *pfmdr1* gene highlight the need for more informative tools, such as genomic wide scan, to identify polymorphisms that could be used as molecular markers of anti-malarial chemoresistance in Brazilian *P. falciparum* isolates.
